# Artificial Intelligence: Knowledge and Attitude Among Lebanese Medical Students

**DOI:** 10.7759/cureus.51466

**Published:** 2024-01-01

**Authors:** Omar A Daher, Ahmad Ayman Dabbousi, Rayan Chamroukh, Abdallah Y Saab, Amir Rabih Al Ayoubi, Pascale Salameh

**Affiliations:** 1 Faculty of Medicine, Beirut Arab University, Beirut, LBN; 2 Department of General Medicine, Faculty of Medical Sciences, Lebanese University, Beirut, LBN; 3 Department of Primary Care and Population Health, University of Nicosia Medical School, Nicosia, CYP; 4 Department of Public Health, Institut National de Santé Publique, d'Épidémiologie Clinique et de Toxicologie (INSPECT-LB), Beirut, LBN; 5 Department of Pharmacy Practice, Lebanese University, Beirut, LBN; 6 School of Medicine, Lebanese American University, Beirut, LBN

**Keywords:** future in medicine, deep learning artificial intelligence, knowledge attitude practices studies, knowledge level, medical school students, artificial intelligence in medicine

## Abstract

Background

Artificial intelligence (AI) has taken on a variety of functions in the medical field, and research has proven that it can address complicated issues in various applications. It is unknown whether Lebanese medical students and residents have a detailed understanding of this concept, and little is known about their attitudes toward AI.

Aim

This study fills a critical gap by revealing the knowledge and attitude of Lebanese medical students toward AI.

Methods

A multi-centric survey targeting 365 medical students from seven medical schools across Lebanon was conducted to assess their knowledge of and attitudes toward AI in medicine. The survey consists of five sections: the first part includes socio-demographic variables, while the second comprises the 'Medical Artificial Intelligence Readiness Scale' for medical students. The third part focuses on attitudes toward AI in medicine, the fourth assesses understanding of deep learning, and the fifth targets considerations of radiology as a specialization.

Results

There is a notable awareness of AI among students who are eager to learn about it. Despite this interest, there exists a gap in knowledge regarding deep learning, albeit alongside a positive attitude towards it. Students who are more open to embracing AI technology tend to have a better understanding of AI concepts (p=0.001). Additionally, a higher percentage of students from Mount Lebanon (71.6%) showed an inclination towards using AI compared to Beirut (63.2%) (p=0.03). Noteworthy are the Lebanese University and Saint Joseph University, where the highest proportions of students are willing to integrate AI into the medical field (79.4% and 76.7%, respectively; p=0.001).

Conclusion

It was concluded that most Lebanese medical students might not necessarily comprehend the core technological ideas of AI and deep learning. This lack of understanding was evident from the substantial amount of misinformation among the students. Consequently, there appears to be a significant demand for the inclusion of AI technologies in Lebanese medical school courses.

## Introduction

Artificial intelligence (AI) is a branch of computer science capable of analyzing complex medical data [1]. It is also defined as a branch of research and engineering concerned with the computational understanding of what is often referred to as intelligent behavior, and the design of machines that display such behavior [2]. In many therapeutic contexts, AI's ability to exploit important relationships within a data collection can be employed in diagnosis, therapy, and outcome prediction [3]. In the medical field, AI has taken on a variety of functions, and research has proven that it can address complicated issues in various applications, particularly in areas where there is a lot of data but no theory [4]. Al may soon surpass doctors in predictive analytics and picture recognition because they cannot process millions of photographs in a reasonable period [5]. Additionally, AI might help choose a drug that lines with the patient's desired course of treatment. Furthermore, scheduling issues or overbooking are other issues that AI may help with [6]. To prevent readmissions, AI could prioritize appointment scheduling depending on the risk of readmission and the overall severity of an illness [7]. The general public has embraced AI because it enables a 4P model of medicine (predictive, preventive, personalized, and participatory) and, consequently, patient autonomy in previously impossible ways [7]. In the cognitive domain, AI can be superior to human intelligence: efficient processing of large amounts of data, memorization, computational capacity, and faultless application of established logical principles [8]. However, it lacks the other fundamental components of our intelligence: consciousness, empathy, compassion, subjectivity, intuition, and the ability to adapt to unique conditions [9].

The hype surrounding AI has led to concerns that it might render certain medical professions, such as radiology, obsolete. However, this is a misconception. In reality, more AI applications are expected to be integrated into medical workflows soon. If these applications generate abrupt alterations to healthcare's contextual integrity and relational dynamics, they could cause substantial ethical and legal difficulties [10]. A key concern with AI is its potential to make incorrect decisions, necessitating the integration of critical thinking as a fundamental aspect of its decision-making processes [11]. A critical point is that physicians should be prepared to alleviate any concerns, uncertainty, or questions that patients and the general public may have when using AI technology. They also have the responsibility to ensure that AI is applied in a manner that enhances patient care [12]. These factors make acquiring solid AI knowledge and expertise a critical responsibility for medical students [13]. Although there is no widely accepted definition or framework for medical AI and its components in existing literature [14], most AI advancements in healthcare predominantly cater to the needs of high-income countries (HICs), where the bulk of this research is conducted [15]. In low- and middle-income countries (LMICs), where personnel shortages and restricted resources limit access to quality care, little has been discussed about what AI may bring to medical practice [16]. It is unknown whether Lebanese medical students and residents have a detailed understanding of this concept, and little is known about their attitudes toward Al and deep learning. To bridge this knowledge gap, a multi-centric survey was undertaken across various medical faculties. This survey aimed to evaluate their attitudes, knowledge, and readiness for AI in medicine and to explore their concerns about AI potentially replacing physicians.

## Materials and methods

The cross-sectional study involved Lebanese medical students enrolled in seven distinct medical institutions across Lebanon: the American University of Beirut, Lebanese American University, Lebanese University, Beirut Arab University, University of Balamand, Holy Spirit University of Kaslik, and Saint Joseph University. Data collection occurred in 2021 and covered diverse regions such as Mount Lebanon, Bekaa, Beirut, and the North and South governments. The study received ethical approval from the Lebanese Hospital Geitawi-University Medical Center (LHG-UMC) Institutional Review Board (IRB# 2021-IRB-017). Prior to the commencement of the study, informed consent was obtained from each participant, with an emphasis on the voluntary nature of their participation. It was explicitly communicated to all participants that their involvement was entirely optional. The survey maintained confidentiality and did not use patient identifiers for the information collected.

Study questionnaire

The survey questionnaire, derived from three previously published studies [17-19], underwent a rigorous validation process. Its validity and reliability were established through a multi-step validation system involving feedback from students and an expert panel of professionals. The Cronbach's alpha for the knowledge and attitude scales were 0.95 and 0.63, respectively, while the Cronbach's alpha for the Medical AI Readiness Scale for Medical Students (MAIRS-MS) was 0.877 [17]. The expert panel approved a final version of the survey, which was then piloted with a cohort of 30 medical students. The questionnaire, initially prepared in English, was translated into Arabic using a back-and-forth translation method. It was administered online to participants who were willing. The survey consisted of five sections: the first addressed socio-demographic variables; the second incorporated the MAIRS-MS [17]; the third explored attitudes toward AI in medicine; the fourth assessed understanding of deep learning; and the fifth focused on considerations regarding radiology as a specialization. Participants were categorized into two groups, 'ready' and 'not ready', based solely on their MAIRS-MS survey scores, using a 5-point Likert scale. These groups were then compared in terms of their attitudes and knowledge.

Statistical analysis

The collected data were entered into Microsoft Excel and analyzed using SPSS v.25 (IBM Corp., Armonk, NY, USA). A p-value of <0.05 was considered statistically significant. Descriptive statistics were conducted, with continuous variables expressed as mean ± SD and categorical variables presented as numbers and percentages. For the bivariate analysis, the independent samples t-test was utilized to test mean differences between two groups, while one-way ANOVA was employed to compare means among more than two groups. Multiple regressions were conducted, considering knowledge and attitude as dependent variables, respectively. Sociodemographic characteristics served as independent variables, with the ENTER method being adopted. Results are presented in terms of the beta coefficient, 95% CI, and p-value.

## Results

A total of 365 Lebanese medical students responded to the questionnaire. Among them, 235 are ready to introduce AI into medicine, while 130 are not. There is no significant difference between genders in terms of readiness to adopt AI in medicine (p=0.443). Awareness of the AI concept is more evident among students who are ready to introduce AI technology (p=0.001). Furthermore, a higher percentage of students from Mount Lebanon (71.6%) are set to use AI compared to those from Beirut (63.2%) (p=0.03). The Lebanese University and Saint Joseph University have the highest number of students willing to incorporate AI into the medical field (79.4% and 76.7%, respectively; p=0.001) (Table 1).

**Table 1:** Demographic distribution of students depending on their willingness to work with AI. AI: Artificial Intelligence.

Variable	Ready, N (%) (n=235)	Not Ready, N (%) (n=130)	P-value
Gender			0.443
Male (n=166)	103 (62.0%)	63 (38.0%)	
Female (n=199)	132 (66.3%)	67 (33.7%)	
Awareness			< .001
Aware (n=300)	211 (70.3%)	89 (29.7%)	
Not Aware (n=65)	24 (36.9%)	41 (63.1%)	
Governate			0.03
Beirut (n=53)	33 (63.2%)	20 (37.7%)	
Mount Lebanon (n=162)	116 (71.6%)	46 (28.4%)	
Other regions (n=150)	86 (57.3%)	64 (42.7%)	
Marital Status			0.454
Single (n=347)	225 (64.8%)	122 (35.2%)	
Other (n=18)	10 (55.6%)	8 (44.4%)	
Parents as physicians			0.431
One or both parents (n=51)	30 (58.8%)	21 (41.2%)	
None (n=314)	205 (65.3%)	109 (34.7%)	
University level			0.288
1st year (n=39)	28 (71.8%)	11 (28.2%)	
2nd year (n=71)	48 (67.6%)	23 (32.4%)	
3rd year (n=34)	27 (79.4%)	7 (20.6%)	
4th year ( n=78)	47 (60.3%)	31 (39.7%)	
5th year (n=50)	28 (56.0%)	22 (44.0%)	
6th year (n=67)	40 (59.7%)	27 (40.3%)	
7th year (n=26)	17 (65.4%)	9 (34.6%)	
Medical School			< .001
American University of Beirut (n=26)	12 (46.2%)	14 (53.8%)	
University of Balamand (n=22)	9 (40.9%)	13 (59.1%)	
Beirut Arab University (n=103)	64 (62.1%)	39 (37.9%)	
Lebanese American University (n=21)	5 (23.8%)	16 (76.2%)	
Lebanese University (n=68)	54 (79.4%)	14 (20.6%)	
Holy Spirit University of Kaslik (n=52)	35 (67.3%)	17 (32.7%)	
Saint Joseph University (n=73)	56 (76.7%)	17 (23.3%)	
Age, mean	21.23 (n=235)	21.83 (n=130)	0.007

Concerning the students' cognition, a significant portion of them cannot define the basic concepts and terminology of AI (45.8%), and half are unable to explain how AI systems are trained (50.2%). However, they are capable of expressing the importance of data collection, analysis, evaluation, and safety for the development of AI in healthcare (53.7%). Regarding their ability factors, one-third of the students are not proficient in using AI effectively and efficiently in healthcare delivery (33.7%), yet they can elucidate how AI applications offer solutions to problems in the healthcare field (44.1%). The majority find AI valuable for use in education services and research purposes (74.8%). Approximately half of the students can articulate the limitations (50.6%), the strengths and weaknesses (51.5%), and the opportunities and threats of AI (57.0%). In the ethical domain, 56.7% of medical students are able to use health data in accordance with legal and ethical norms, and 51.7% can follow legal regulations while using AI technologies (Table 2).

**Table 2:** Assessment of the self-reported technology comprehension. AI: Artificial Intelligence.

Item	Strongly Agree/Agree, N (%)	Neutral, N (%)	Strongly Disagree/Disagree, N (%)
Cognition Factor			
I can define the basic concepts of data science	168 (46.0)	116 (31.8)	81 (22.2)
I can define the basic concepts of statistics	270 (73.9)	55 (15.1)	40 (11.0)
I can explain how AI systems are trained	64 (17.5)	118 (32.3)	183 (50.2)
I can define the basic concepts and terminology of AI	96 (26.3)	102 (27.9)	167 (45.8)
I can properly analyze the data obtained by AI in healthcare	78 (21.4)	119 (32.6)	168 (46.0)
I can differentiate the functions and features of AI-related tools and applications	75 (20.5)	104 (28.5)	186 (50.9)
I can organize workflows compatible with AI	59 (16.1)	118 (32.3)	188 (51.5)
I can express the importance of data collection, analysis, evaluation, and safety; for the development of AI in healthcare	196 (53.7)	88 (24.1)	81 (22.2)
Ability Factor			
I can harness AI-based information combined with my professional knowledge	147 (40.3)	108 (29.6)	110 (30.2)
I can use AI technologies effectively and efficiently in healthcare delivery	120 (32.9)	122 (33.4)	123 (33.7)
I can use AI applications in accordance with their purpose	150 (41.1)	108 (29.6)	107 (29.3)
I can access, evaluate, use, share and create new knowledge using information and communication technologies	180 (49.3)	103 (28.2)	82 (22.5)
I can explain how AI applications offer a solution to which problem in healthcare	161 (44.1)	106 (29.0)	98 (26.9)
I find it valuable to use AI for education, service, and research purposes	273 (74.8)	65 (17.80.0)	27 (7.4)
I can explain the AI applications used in healthcare services to the patient	140 (38.3)	106 (29.0)	119 (32.6)
I can choose a proper AI application for the problem encountered in healthcare	100 (27.4)	119 (32.6)	146 (40.0)
Vision Factor			
I can explain the limitations of AI technology	185 (50.6)	96 (26.3)	84 (23.1)
I can explain the strengths and weaknesses of AI technology	188 (51.5)	93 (25.5)	84 (23.0)
I can foresee the opportunities and threats that AI technology can create	208 (57.0)	92 (25.2)	65 (17.8)
Ethics Factor			
I can use health data in accordance with legal and ethical norms	207 (56.7)	96 (26.3)	62 (17.0)
I can conduct under ethical principles while using AI technologies	182 (49.9)	119 (32.6)	64 (17.5)
I can follow legal regulations regarding the use of AI technologies in healthcare	189 (51.7)	113 (31.0)	63 (17.3)

Attitude

Regarding students' attitudes towards AI, most of them do not believe that physicians will be replaced by AI in the future (75.3%). However, they do believe that AI will assist them (87.9%). Furthermore, 96.2% think that all medical students should receive teaching in the AI field. They believe that this will make medicine more exciting (72.6%) (Table 3).

**Table 3:** Attitudes toward AI. AI: Artificial Intelligence.

Item	Answers	N	%
General physicians will be replaced in the foreseeable future	Agree	90	24.7
	Disagree	275	75.3
All medical students should receive teaching in AI	Agree	351	96.2
	Disagree	14	3.8
These developments in AI make medicine more ________	Exciting	265	72.6
	Frightening	100	27.4
AI will ________ physicians	Assist	321	87.9
	Displace	44	12.1

Knowledge

Regarding their knowledge, a significant majority of Lebanese medical students lack a clear understanding of the definition, methods, and application of deep learning, with 77.8%, 84.7%, and 62.7% providing incorrect answers, respectively (Table 4). Delving into a more specific aspect, only 26.8% of medical students consider radiology as a residency option, and among these, 60.2% cited AI as the reason for their choice.

**Table 4:** Knowledge of deep learning.

Item	True, N (%)	False/I don't know, N (%)
Deep learning is a class of machine learning algorithms that use multiple layers of neural networks	81 (22.2)	284 (77.8)
Deep learning methods learn directly from data without the need for hand-engineered feature extraction	56 (15.3)	309 (84.7)
The application of deep learning in radiology requires large databases of labeled medical images	136 (37.3)	229 (62.7)
Deep learning systems are often opaque; delineating their underlying 'thought process' can be difficult	57 (15.6)	308 (84.4)
Existing deep learning technology achieves good pattern recognition but lacks deductive reasoning ability	85 (23.3)	280 (76.7)

Factors associated with knowledge and attitudes

Multiple regressions were conducted with knowledge and attitude as the dependent variables, respectively. Sociodemographic variables served as independent variables, and the ENTER method was used. Results are presented in terms of the beta coefficient, 95% CI, and p-value. Participants from the Lebanese American University (p=0.028), those with parents who are doctors (p<0.05), and female participants (p=0.001) demonstrated significantly lower knowledge compared to other participants. Conversely, first-year students exhibited higher knowledge (p=0.013), as did those from Jbeil-Kesrouan (p=0.054) and those who are engaged (p=0.004) (Table 5).

**Table 5:** Factors associated with knowledge of AI. AI: Artificial Intelligence; B: Beta Coefficient.

Parameter	B	95% Wald Confidence Interval for Exp(B)		P-value
		Lower	Upper	
University				
American University of Beirut	-0.010	0.494	1.984	0.978
University of Balamand	-0.235	0.394	1.585	0.508
Beirut Arab University	-0.065	0.579	1.516	0.792
Lebanese American University	-0.834	0.207	0.913	0.028
Holy Spirit University of Kaslik	-0.287	0.431	1.307	0.311
Saint Joseph University	0.011	0.621	1.648	0.964
Lebanese University = Reference				
University level				
1st year	1.336	1.330	10.878	0.013
2nd year	0.117	0.445	2.840	0.804
3rd year	0.188	0.484	3.007	0.687
4th year	-0.098	0.432	1.903	0.795
5th year	0.068	0.511	2.246	0.856
6th year	0.205	0.629	2.394	0.548
7th year = Reference				
Governate				
Akkar	-0.310	0.223	2.415	0.610
Baalbek-Hermel	-0.494	0.229	1.627	0.323
Beirut	0.078	0.587	1.989	0.803
Beqaa	0.363	0.733	2.823	0.291
Jubail-Keserwan	0.848	0.987	5.522	0.054
Mount Lebanon	0.191	0.715	2.046	0.477
Nabatieh	-0.052	0.399	2.257	0.907
North Lebanon	0.030	0.551	1.926	0.925
South Lebanon = Reference				
Gender				
Female versus Male	-0.514	0.443	0.808	0.001
Parents as physicians				
My father only is a doctor	-0.734	0.173	1.330	0.158
My mother only is a doctor	-1.611	0.045	0.884	0.034
No, neither my father nor my mother is a doctor	-0.905	0.166	0.985	0.046
Yes, both of my parents are doctors = Reference				
Marital status				
Engaged	1.689	1.732	16.918	0.004
Married	0.163	0135	10.282	0.883
Other	0.115	0.436	2.882	0.812
Single = Reference				

Being engaged was significantly associated with a more positive attitude towards AI (p=0.003), whereas being married correlated with a more negative attitude (p=0.037). Additionally, a more positive attitude about AI was also associated with higher knowledge (p=0.005) (Table 6).

**Table 6:** Factors associated with attitudes toward AI. AI: Artificial Intelligence; B: Beta coefficient.

Parameters	B	95% Wald Confidence Interval		P-value
		Lower	Upper	
Marital Status				
Engaged	0.806	0.281	1.331	0.003
Married	-1.050	-2.037	-0.063	0.037
Other	-0.270	-0.700	0.159	0.218
Single = Reference				
Knowledge of AI	0.067	0.020	0.114	0.005

## Discussion

This study reports the first-ever survey on the perception of AI involving a large group of Lebanese medical students from various universities. Based on the collected data, there is a high awareness of AI among students who are eager to learn about it. However, there is a notable lack of knowledge about deep learning despite a generally positive attitude towards it. This finding aligns with a similar study where approximately 76% of healthcare students expressed a positive and optimistic attitude towards the incorporation of AI in clinical practice and its potential future applications [20]. AI and deep learning are poised to significantly impact the future of radiology and medicine in general [13]. This topic, introduced in 1956, has recently become a focus for new researchers, especially radiologists, and is a main topic at several conferences [17]. The primary concern has been whether radiologists will lose their jobs due to these technologies and if radiology as a specialty might become redundant. These approaches have mostly been used for visual tasks, such as image classification (e.g., chest X-ray diagnosis), or automated segmentation of regions of interest in an image (e.g., segmentation of tumor tissue in brain MRI) [21]. Major news outlets have also discussed these issues, with some articles suggesting that algorithms might eventually surpass human radiologists in detecting pneumonia [22]. However, the survey revealed a significant number of students considering radiology as a future specialty, who believe AI will not only make medicine more exciting but also enhance the speed and accuracy of our work, thereby improving efficiency in our daily lives. Despite anecdotal reports suggesting that some students are hesitant to pursue radiology due to fears about AI, the majority of Lebanese students are optimistic, believing that human physicians and radiologists will continue to be essential in the future. This sentiment aligns with recent findings among Jordanian students, who also largely believe that AI is unlikely to replace healthcare professionals in most tasks anytime soon [23].

In the study, it was concluded that most medical students may not fully grasp the core technological concepts of AI and deep learning. A similar trend was noted in a study conducted in Saudi Arabia [14]. This lack of understanding was evident from the substantial amount of incorrect information held by students. The knowledge gap is attributed to AI being categorized as computer science, a field considered distant from traditional medical studies and developed primarily within that sphere [24]. This categorization explains the significant gap in AI knowledge among Lebanese medical students, stemming from a lack of comprehensive AI courses in their educational curriculum, and considering the fast evolution and the big overflow of information in this field, Lebanese medical students seem to be less advanced in this category compared to other countries where AI makes the cornerstone of medicine in general and radiology specifically [1]. On the other hand, the students believe in AI, and they consider that all medical students should receive teaching about it, as it will make medicine more exciting and easy. It was rumored that doctors, especially radiologists, would be frightened.

Talking about all factors of AI, there is a promising cognitive ability among Lebanese medical students as they can define the basic concepts but cannot apply them, and they can use technology effectively and efficiently in healthcare units. Students appreciate the significant role of AI in research, in saving time, and in producing the most accurate results. AI tools have the capacity to assist researchers in various aspects of their work, including gathering pertinent research articles, pinpointing gaps in existing literature, devising research inquiries, suggesting suitable statistical methods for available data, generating graphical representations of data, and aiding in manuscript writing [25]. From an ethical perspective, they seem to understand the ethical standards and boundaries while using patient data and are aware of the legal consequences that can follow violations. Consider the massive volumes of data that AI can access, ranging from genetic, biomarker, and phenotypic data to health records and delivery systems. Decisions in data-intensive fields like radiology, pathology, and ophthalmology are already aided by technology [17]. Considering the vast amount of ambiguity about AI in the population, students should have a strong understanding of all AI applications so they can inform patients about their privacy and the accuracy of the results, thereby alleviating any doubts in decision-making. The method of introducing AI into medicine in Lebanon is not yet clear; it could take several forms and may be introduced to some societies before others. However, interest in AI is widespread across all universities and regions in Lebanon. Therefore, based on these data, we should embrace the revolution and work on integrating these technologies into our educational systems, considering the optimistic view toward AI and the willingness to use these technologies to enhance the quality of care provided to patients. Finally, incorporating AI into the medical curriculum could be challenging, as it would need to comply with university regulations and national accrediting organizations [26]. However, a study indicated that AI could help automate numerous administrative tasks, thereby improving overall efficiency [27]. Furthermore, a broader study focusing on the perspectives of academic staff, university administration, and accrediting authorities toward the value and feasibility of implementing AI in core education would provide more relevant insights [28].

It is important to acknowledge that this study has certain limitations in terms of its population representation, as the distribution of students across universities may not accurately mirror that of the broader society. Additionally, the gender distribution, governorate ratio, and student participation rate may not faithfully depict the demographics of Lebanese medical students, introducing some selection bias. There is also the potential for information bias, where questions could be misunderstood, leading to random responses from participants. Furthermore, confounding bias may be present, possibly overlooking some confounding factors in the multivariate analysis. Moreover, the study exclusively focused on the attitudes of undergraduate medical students, neglecting postgraduate students and more experienced doctors' perspectives. Exploring these diverse groups and addressing their specific concerns could be an intriguing avenue for future research. Lastly, regarding the correlation with specialty, it is worth noting that the discussion of AI could extend beyond radiology to various other medical specialties.

## Conclusions

In medicine, AI is playing an increasingly important role. It is a rapidly developing topic with many applications in various fields. AI is seen as a collaborator, not a competitor, and it is not viewed as a threat to radiologists. These technologies are positively regarded by the next generation of Lebanese medical students, who, despite lacking a deep understanding of these technologies and their applications, hope to incorporate them into their future practice. AI is often underestimated in developing countries, particularly in Lebanon, where comprehensive studies assessing the readiness of medical students to use deep learning technology are lacking. As a result, there may be significant demand for the inclusion of AI technologies in university curricula, and for further exploration through articles or conferences, which warrants additional investigation.
